# Highly pathogenic avian influenza A(H5N1) virus infection in foxes with PB2-M535I identified as a novel mammalian adaptation, Northern Ireland, July 2023

**DOI:** 10.2807/1560-7917.ES.2023.28.42.2300526

**Published:** 2023-10-19

**Authors:** Paula Lagan, Robyn McKenna, Salam Baleed, Bob Hanna, Jason Barley, Shirley McConnell, Anastasia Georgaki, Tarja Sironen, Ari Kauppinen, Tuija Gadd, Erika Lindh, Niina Ikonen, Michael J McMenamy, Ken Lemon

**Affiliations:** 1 Veterinary Sciences Division, Agri-Food and Biosciences Institute (AFBI) Belfast, Northern Ireland; 2 Veterinary Service Animal Health Group, Department of Agriculture, Environment and Rural Affairs (DAERA), Ballykelly, Northern Ireland; 3 Department of Veterinary Biosciences, University of Helsinki, Helsinki, Finland and Medicum, University of Helsinki, Helsinki, Finland; 4 Finnish Food Authority (Ruokavirasto), Helsinki, Finland; 5 Department of Health Security, The Finnish Institute for Health and Welfare (THL), Helsinki, Finland

**Keywords:** Highly pathogenic avian influenza virus, H5N1, mammalian infection, clade 2.3.4.4b, HPAI, BB genotype

## Abstract

We report cases of mammalian infection with highly pathogenic avian influenza (HPAI) virus A(H5N1) clade 2.3.4.4b in Northern Ireland. Two common gulls (*Larus canus*) and two red fox kits (*Vulpes vulpes*), were found dead in close vicinity. Comparison of viral whole genome sequences obtained from the animals identified a novel mammalian adaptation, PB2-M535I. Analysis of genetic sequences from other recent mammalian infections shows that this mutation has arisen on at least five occasions in three European countries since April 2023.

The global spread of highly pathogenic avian influenza (HPAI) A(H5Nx) clade 2.3.4.4b has resulted in an unprecedented number of spillover events to wild and domestic mammals, leading to public health concerns over the increased zoonotic risks posed [1,2]. Here, we report the first confirmed cases of mammalian infection with HPAI A(H5N1) clade 2.3.4.4b in Northern Ireland, affecting two red foxes. We present analysis of the viral sequences obtained from the foxes, and two epidemiologically linked avian sequences, and identify a novel mammalian adaptation.

## Event detection

**Figure 1:**
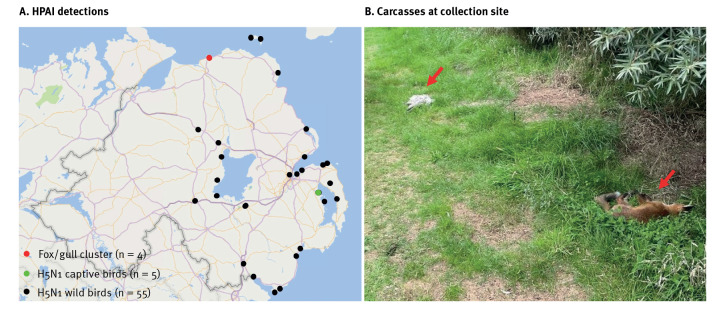
(A) Locations of all highly pathogenic avian influenza detections, October 2022–August 2023 (n = 64 detections at 30 locations) and (B) Fox and gull carcasses at the collection site, Northern Ireland, July 2023 (n = 4)

On 25 July 2023, the Department of Agriculture, Environment and Rural Affairs (DAERA) was notified of two gulls and two red foxes found dead in sand dunes located on the north coast of Northern Ireland (Figure 1A and 1B). Given the unprecedented spread of HPAI in wild seabirds and findings of spillover to mammalian species, DAERA decided to investigate this event as part of passive surveillance. Veterinary officers attended the scene and confirmed that there was no human exposure to the infected animals. All four carcasses were collected by trained staff wearing appropriate personal protective equipment, packaged and transported following the competent authority's protocols, and delivered to the Agri-Food and Bioscience Institute (AFBI) for post-mortem examination and diagnostic testing. No further action was taken at the site of collection.

HPAI: highly pathogenic avian influenza.

## Virological analysis

Tissue samples including brain, lung/trachea, intestines and visceral organs from all animals along with nasal and oropharyngeal swabs from foxes were collected, processed and total RNA extracted. Following the standard testing algorithm for avian influenza virus at AFBI, samples were initially tested for the presence of influenza A virus matrix (M) gene RNA by real-time reverse transcription PCR (rRT-PCR) as described previously [3]. All samples tested positive, with the strongest detection of viral RNA in brain samples taken from the foxes (Table 1). Additional subtyping/pathotyping rRT-PCR assays were performed as described previously, and confirmed that all samples were positive for HPAI A(H5N1) [4,5].

**Table 1:** Detection of avian influenza virus in tissue/swab samples from foxes and gulls, Northern Ireland, July 2023 (n = 4)

Sample source	Sample type	Avian influenza virus rRT-PCR results					
		M gene		HP H5 gene		N1 gene	
		Ct value	Result	Ct value	Result	Ct value	Result
Fox 1	Nasal swab^a^	24.02	Positive	24.86	Positive	25.53	Positive
Fox 2	Nasal swab^a^	25.58	Positive	27.02	Positive	27.39	Positive
Fox 1	Oropharyngeal swab	24.12	Positive	25.78	Positive	27.22	Positive
Fox 2	Oropharyngeal swab	24.77	Positive	26.19	Positive	26.93	Positive
Fox 1 and 2 (pooled)	Brain	18.73	Positive	20.01	Positive	20.77	Positive
Fox 1 and 2 (pooled)	Respiratory tissues	21.98	Positive	21.13	Positive	21.83	Positive
Fox 1 and 2 (pooled)	Intestines	28.48	Positive	26.67	Positive	28.68	Positive
Fox 1 and 2 (pooled)	Viscera	26.13	Positive	23.91	Positive	25.26	Positive
Gull 1	Respiratory tissues^a^	28.03	Positive	28.46	Positive	29.52	Positive
Gull 2	Respiratory tissues^a^	21.07	Positive	21.78	Positive	22.64	Positive
Gull 1	Intestines	27.98	Positive	27.93	Positive	29.35	Positive
Gull 2	Intestines	28.04	Positive	28.38	Positive	29.43	Positive
Gull 1	Viscera	28.26	Positive	28.87	Positive	30.13	Positive
Gull 2	Viscera	25.79	Positive	25.73	Positive	25.83	Positive
Gull 1 and 2 (pooled)	Brain	21.70	Positive	23.09	Positive	23.15	Positive

^a^ Samples selected for whole genome sequencing.

## Histopathological examination

**Figure 2:**
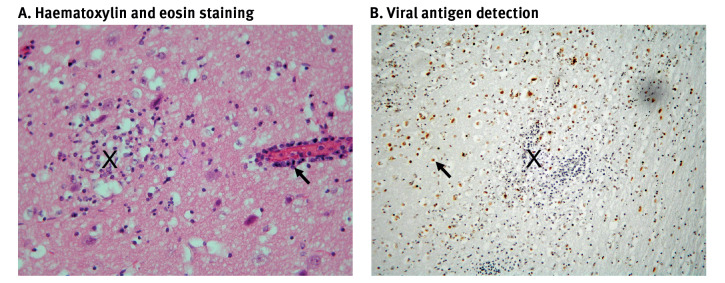
Histopathological examination of lesions and influenza virus antigen distribution in brain tissue from foxes, Northern Ireland, July 2023

Histopathological examination of fox brain sections indicated non-suppurative meningo-encephalitis consistent with viral infection, with associated lymphocytic vasculitis, perivascular lymphocytic cuffing and vascular endothelial necrosis (Figure 2A). The observed lesions were highly similar to those described in a previous study of HPAI A(H5N1) clade 2.3.4.4b infected red foxes in New York state, United States [6]. Immunohistochemical staining using a monoclonal antibody that recognises influenza A virus NP protein (clone EBS-I-238, European Veterinary Laboratory), revealed large numbers of infected cells in the cerebrum (Figure 2B).

A. Histology of brain section illustrating lesions with haematoxylin and eosin-stained section of fox cerebrum showing focal encephalomalacia and spongiosis (X), which occurred mainly in the grey matter, and perivascular lymphocytic cuffing (arrow). There was localised microgliosis in the malacic areas.

B. Immunoperoxidase-labelled section showing NP reactivity of the influenza A virus in the vicinity of microglial foci in the cerebrum (X), with positive signalling in the nuclei in particular (arrow), as would be expected for a nucleotrophic virus. Nuclear karyorrhexis was a feature in the affected areas.

## Whole genome sequencing and phylogenetic analysis

**Figure 3:**
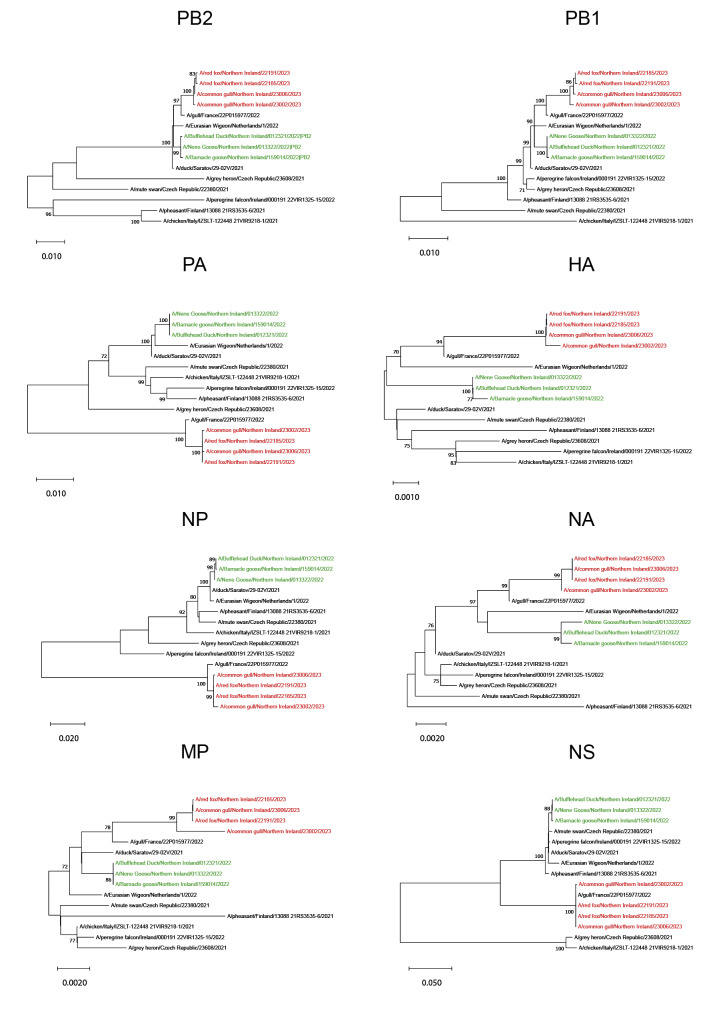
Phylogenetic analysis of highly pathogenic avian influenza A(H5N1) virus sequences collected from gulls and foxes, Northern Ireland, July 2023 (n = 4) compared with captive bird sequences, Northern Ireland, October 2022 (n = 3) and clade 2.3.4.4b sequences collected in Europe, 2021–2023 (n = 8)

We performed amplicon-based whole genome sequencing (WGS) on RNA from selected samples (Table 1), as described previously [7]. Phylogenetic analysis of WGS data indicated that the viral sequences obtained from the gull and fox samples belonged to the BB genotype of clade 2.3.4.4b (H5N1-A/gull/France/22P015977/2022-like; Figure 3; Supplementary Table S1 provides details of sequences used in the phylogenetic analysis). Since April/May 2023, this genotype has accounted for ca 90% of all detections in Europe, the vast majority of which have occurred in gulls [1]. Since June 2023, the majority of HPAI A(H5N1) detections in wild birds in Northern Ireland have involved gulls, especially black-headed gulls.

Individual complete segments of HPAI (A)H5N1 virus PB2, PB1, PA, HA, NP, NA, MP and NS were aligned using MUSCLE in MEGA11 (http://www.megasoftware.net), and maximum likelihood analysis performed, with phylogeny tested using 1,000 bootstrap replications. Fox/gull sequences are highlighted in red, other avian sequences from Northern Ireland in green and representatives of all common recent European genotypes in black.

Phylogenetic analysis of WGS derived from gull and fox samples showed that both fox sequences were highly similar and closely related to the Gull 2 sequence (A/common gull/Northern Ireland/23006/2023; Figure 3; Supplementary Table S2 provides an analysis of the nucleotide variation between fox- and gull-derived HPAI (A)H5N1 sequences). At the nucleotide level, the Gull 2 sequence differed by only 5 (vs Fox 1: A/red fox/Northern Ireland/22185/2023) or 7 (vs Fox 2: A/red fox/Northern Ireland/22191/2023) positions. The Gull 1 sequence (A/common gull/Northern Ireland/23002/2023) was less closely related to the fox sequences, differing by 21 or 23 nucleotide positions, respectively. We therefore propose that the most likely scenario involves both foxes becoming infected via contact with Gull 2, although we cannot exclude the possibility that infection was from another avian source. We speculate that the fox den was located close to the collection site, which may explain the distribution of fox and gull carcasses at this location.

## Identification of candidate mammalian adaptations

We compared the amino acid sequences from the fox/avian coding regions to identify potential mammalian adaptations. Mutations at three locations were unique to the fox sequences, with the Fox 1 sequence bearing PB2-T271A and the Fox 2 sequence bearing PB2-M535I and PB1-F2-T7I (Table 2).

**Table 2:** Amino acid variation between fox and gull-derived highly pathogenic avian influenza (A)H5N1 virus sequences, Northern Ireland, July 2023 (n = 4)

Sample source	Sequence	Amino acid mutations										
		PB2		PB1	PB1-F2		NP	NA	M1		M2	NS1
		271	535	187	7	63	402	193	115	187	23	225
Fox 1	A/red fox/Northern Ireland/22185/2023	**A**	M	R	T	F	S	V	I	K	S	T
Fox 2	A/red fox/Northern Ireland/22191/2023	T	**I**	R	**I**	F	S	V	I	K	S	T
Gull 2	A/common gull/Northern Ireland/23006/2023	T	M	**K**	T	F	S	V	I	K	S	T
Gull 1	A/common gull/Northern Ireland/23002/2023	T	M	R	T	**S**	**K**	**I**	**V**	**R**	**N**	**A**

Variant residues are indicated in bold.

Since October 2022, PB2-T271A has been frequently identified in mammalian isolates of H5N1 clade 2.3.4.4b viruses and, along with PB2-E627K and PB2-D701N, is associated with increased virulence and replication in mammals [1,8].

## Discussion

In Northern Ireland, detection of the first case of HPAI A(H5N1) during the 2022–23 epidemiological year occurred in October 2022 in wild and captive *Anatidae* species (ducks, geese and swans) on a large wetland reserve. The WGS analysis identified the AB genotype (H5N1-A/duck/Saratov/29–02/2021-like). Over the winter months and into spring 2023, a small number of detections were observed in various wild bird species including geese, swans and raptors. From early June to late July 2023, a significant increase in detections occurred, with 14 cases involving 36 seabirds of the *Laridae* family (black-headed gulls, kittiwakes and terns) found dead around the northern and eastern coastal regions. While WGS was not performed for these cases, it seems likely that the epidemic in Northern Ireland has proceeded as elsewhere in Europe, with the AB genotype dominating at the start of the epidemiological year, followed by the rise of genotype BB in the latter half, with subsequent spillover to mammalian species.

Interspecies transmission of HPAI to mammals is rare. However, the current panzootic caused by HPAI A(H5N1) virus has witnessed unprecedented numbers of mammalian spillover events in both the Americas and Europe [1]. This observation has raised concerns that the virus may eventually adapt to mammalian hosts and become capable of sustained mammal-to-mammal spread. Widespread surveillance, including genetic characterisation of mammalian detections, is therefore critical to monitor HPAI A(H5N1) virus evolution.

A major barrier to interspecies transmission is the inability of avian-origin viral polymerases to carry out efficient replication in mammalian cells. Approximately half of all mammalian viruses from the current panzootic characterised to date contain at least one of three mutations that enable the replicative ability of the viral polymerase in mammalian cells [1]. The most commonly observed is PB2-E627K. In the absence of PB2-E627K, the mutations PB2-T271A and PB2-D701N are also thought confer similar functionality. A recent outbreak of HPAI A(H5N1) in domestic cats in Poland identified PB2-K526R in combination with PB2-E627K [9]. PB2-K526R is a known mammalian adaptation thought to enhance the effect of PB2-E627K [10].

In our current study, we identify PB2-M535I as a newly emerging mammalian adaptation of clade 2.3.4.4b viruses. The close epidemiological link between the infected gulls and foxes and the genetic similarity of the viral sequences allows the observed differences between the avian and mammalian sequences to be assessed with high confidence. Further in vivo/in vitro studies are needed to clearly define the effect of this mutation on the biological characteristics of the virus.

The mutation PB2-M535I has not been previously described as a mammalian adaptation during the current global outbreak of HPAI A(H5Nx) clade 2.3.4.4b viruses. Analysis of multiple human cases of influenza A(H7N9) virus infection, which first emerged in eastern China in 2013, identified PB2-M535L as one of three mutations (along with PB2-Q591K and PB2-D701N) that could restore viral polymerase activity in mammalian cells in the absence of PB2-E627K [11]. Methionine at position 535 of PB2 is completely conserved in European avian H5N1 clade 2.3.3.4b strains. Examination of all available mammalian sequences shows that PB2-M535I has emerged on four other occasions in addition to the case described here. It was first detected in a fox, collected on 7 April 2023 in the Rovigo province of Italy (A/fox/Italy/23VIR3885–1/2023 EPI_ISL_17679728). Since 14 July 2023, a major outbreak of H5N1 has been ongoing in farmed foxes, minks and racoon dogs on a large number of fur farms in the South and Central Ostrobothnia region of Finland [12]. A subset of WGS from this outbreak showed canonical PB2 mammalian adaptations (PB2-E627K and PB2-T271A). However, we noted that PB2-M535I occurred in at least three animals: A/arctic-fox/Finland/621/2023 EPI_ISL_18122439 (collected 24 July 2023), A/blue fox/Finland/2023AI06820 015/2023 EPI_ISL_18131276 (collected 3 August 2023) and A/silver fox/Finland/2023AI06834 029/2023 EPI_ISL_18131279 (collected 4 August 2023). Taken together, we propose PB2-M535I as a newly emerged mammalian adaptation.

The PB1-F2-T7I substitution has not been previously described as a mammalian adaptation. PB1-F2-T7 is highly conserved in both avian and mammalian derived H5Nx clade 2.3.4.4b European sequences, with PB1-F2-I7 occurring at a frequency of 1.2% and 2.1%, respectively (data not shown). Therefore, although the available data indicate that PB1-F2-T7I emerged during replication within the fox host, there is insufficient evidence to suggest that this is an authentic mammalian adaptation.

The two fox-derived sequences differ at only three nucleotide positions, strongly suggesting a common source of infection, with Gull 2 or another closely related avian source most likely. The distinct patterns of mammalian adaptation displayed (PB2-T271A vs PB2-M535I) also suggest that these mutations arose independently during replication within each fox host rather than via mammal-to-mammal spread.

## Conclusion

Analysis of an epidemiologically linked cluster of HPAI A(H5N1) virus infected foxes and gulls has identified PB2-M535I as a recently emerged mammalian adaptation of clade 2.3.4.4b viruses. Retrospective analysis of submitted genetic sequences shows that this mutation has arisen on at least five occasions in three European countries since April 2023. Surveillance efforts should monitor future mammalian derived sequences for this mutation in addition to the other well characterised adaptations.

## Supplementary Data

128000 Supplement
