# Supplementary material for: Highly pathogenic avian influenza A(H5N1) virus infection in foxes with PB2-M535I identified as a novel mammalian adaptation, Northern Ireland, July 2023
Source: Euro Surveill. 2023 Oct 19;28(42):2300526. doi: 10.2807/1560-7917.ES.2023.28.42.2300526 (PMC10588307; doi:10.2807/1560-7917.ES.2023.28.42.2300526)
Supplement: Supplement [file 23-00526_LEMON_SUPPLEMENT.pdf]

Supplementary Material

This supplementary material is hosted by *Eurosurveillance* as supporting information alongside the article *Highly pathogenic avian influenza A(H5N1) infection in foxes identified PB2-M535I as a novel mammalian adaptation, Northern Ireland, July 2023*, on behalf of the authors, who remain responsible for the accuracy and appropriateness of the content. The same standards for ethics, copyright, attributions and permissions as for the article apply. Supplements are not edited by *Eurosurveillance* and the journal is not responsible for the maintenance of any links or email addresses provided therein

Supplementary Table S1. Origin of the HPAI sequences used for phylogenetic analysis

Isolate name	Isolate ID	Collection date	Submission date	Originating lab	Submitting lab
A/Nene_Goose/Northern_Ireland/013322/2022	EPI_ISL_15586116	2022-10-10	2022-10-31	Animal and Plant Health Agency (APHA)	Animal and Plant Health Agency (APHA)
A/Barnacle_goose/Northern_Ireland/159014/2022	EPI_ISL_16384152	2022-11-17	2023-01-04	Animal and Plant Health Agency (APHA)	Animal and Plant Health Agency (APHA)
A/Bufflehead_Duck/Northern_Ireland/012321/2022	EPI_ISL_15586107	2022-10-10	2022-10-31	Animal and Plant Health Agency (APHA)	Animal and Plant Health Agency (APHA)
A/Eurasian Wigeon/Netherlands/1/2022	EPI_ISL_15925875	2022-10-29	2022-11-28	Erasmus Medical Center	Erasmus Medical Center
A/duck/Saratov/29-02V/2021	EPI_ISL_5463797	2021-09-30	2021-10-25	Center of Hygiene and Epidemiology in Saratov Oblast	State Research Center of Virology and Biotechnology (VECTOR)
A/mute swan/Czech Republic/22380/2021	EPI_ISL_9603920	2021-11-15	2022-02-07	State Veterinary Institute Prague	State Veterinary Institute Prague
A/chicken/Italy/IZSLT-122448_21VIR9218-1/2021	EPI_ISL_7733644	2021-10-28	2021-12-16	Istituto Zooprofilattico Sperimentale del Lazio e della Toscana "M. Aleandri"	Istituto Zooprofilattico Sperimentale Delle Venezie
A/peregrine_falcon/Ireland/000191_22VIR1325-15/2022	EPI_ISL_11259265	2022-01-05	2022-03-22	Central Veterinary Research Laboratory	Istituto Zooprofilattico Sperimentale delle Venezie
A/pheasant/Finland/13088_21RS3535-6/2021	EPI_ISL_15518266	2021-10-01	2022-10-27	Finnish Food Authority	Istituto Zooprofilattico Sperimentale Delle Venezie
A/grey heron/Czech_Republic/23608/2021	EPI_ISL_8515481	2021-11-28	2022-01-09	State Veterinary Institute Prague	State Veterinary Institute Prague
A/gull/France/22P015977/2022	EPI_ISL_13519451	2022-05-11	2022-06-29	Anses (Ploufragan-Plouzané)	ANSES Agence Nationale De Securite Sanitaire De L'alimentation
A/fox/Italy/23VIR3885-1/2023	EPI_ISL_17679728	2023-04-07	2023-05-18	Istituto Zooprofilattico Sperimentale delle Venezie, EU/OIE/Reference Laboratory and FAO Reference Centre for AI and ND	Istituto Zooprofilattico Sperimentale Delle Venezie
A/arctic-fox/Finland/621/2023	EPI_ISL_18122439	2023-07-24	2023-08-22	Finnish Food Authority	Finnish Institute for Health and Welfare, THL
A/blue fox/Finland/2023AI06820_015/2023	EPI_ISL_18131276	2023-08-03	2023-08-24	University of Helsinki, Department of Virology	Friedrich-Loeffler-Institut
A/silver fox/Finland/2023AI06834_029/2023	EPI_ISL_18131279	2023-08-04	2023-08-24	University of Helsinki, Department of Virology	Friedrich-Loeffler-Institut

Supplementary Table S2. Nucleotide variation between fox and gull derived HPAI (A)H5N1 sequences

Nucleotide differences	PB2					PB1							PA		HA	NP					NA	MP				NS
	838	1431	1632	2028	2067	138	306	584	741	1113	1239	1698	276	2088	13	21	129	1249	1250	1257	597	136	368	585	781	699
A/red fox/Northern Ireland/22185/2023 (Fox 1)	**G**	C	G	C	C	C	T	G	G	G	C	A	A	A	G	T	T	T	C	A	G	C	A	A	G	A
A/red fox/Northern Ireland/22191/2023 (Fox 2)	A	C	**A**	C	C	**T**	T	G	G	G	C	A	A	A	G	T	T	T	C	A	G	C	A	A	G	A
A/common gull/Northern Ireland/23006/2023 (Gull 2)	A	C	G	C	T	C	T	**A**	G	G	C	**G**	A	**G**	G	**C**	T	T	C	A	G	C	A	A	G	A
A/common gull/Northern Ireland/23002/2023 (Gull 1)	A	**T**	G	**T**	**T**	C	**C**	G	**A**	**A**	**T**	**G**	**G**	A	**T**	T	**C**	**A**	**A**	**T**	**A**	**T**	**G**	**G**	**A**	**G**
